# The Cardioprotective Actions of Hydrogen Sulfide in Acute Myocardial Infarction and Heart Failure

**DOI:** 10.1155/2014/768607

**Published:** 2014-06-22

**Authors:** David J. Polhemus, John W. Calvert, Javed Butler, David J. Lefer

**Affiliations:** ^1^Department of Pharmacology and Experimental Therapeutics and Cardiovascular Center of Excellence, LSU Health Sciences Center, New Orleans, LA 70112, USA; ^2^Department of Surgery, Emory University School of Medicine, Atlanta, GA 30322, USA; ^3^Department of Medicine, Emory University School of Medicine, Atlanta, GA 30322, USA

## Abstract

It has now become universally accepted that hydrogen sulfide (H_2_S), previously considered only as a lethal toxin, has robust cytoprotective actions in multiple organ systems. The diverse signaling profile of H_2_S impacts multiple pathways to exert cytoprotective actions in a number of pathological states. This paper will review the recently described cardioprotective actions of hydrogen sulfide in both myocardial ischemia/reperfusion injury and congestive heart failure.

## 1. Introduction 

**Figure 1:**
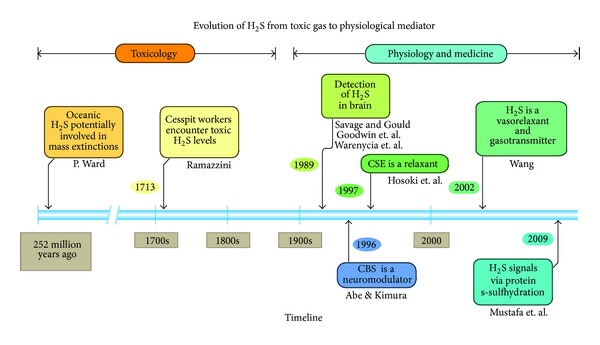
History of the emergence of hydrogen sulfide (H_2_S) as a physiological regulator of cardiovascular homeostasis. H_2_S is believed to be responsible for mass extinctions that occurred over 250 million years ago as toxic gases were spewed from deep in the earth. In the 1700s, H_2_S was linked to injuries sustained by sewer workers. In 1989, H_2_S was detected in the brain of mammals by several groups. In 1996-1997, H_2_S was shown to modulate vascular tone and neuronal function. Finally in 2002, H_2_S was implicated in vascular function and blood pressure regulation in seminal studies. H_2_S was then shown to posttranslationally modify proteins via s-sulfhydration by Dr. Sol Snyder's group. Adopted from Hideo Kimura, Ph.D. Ward [71], Savage and Gould [72], Goodwin et al. [73], Warenycia et al. [74], and Mustafa et al. [75].

Hydrogen sulfide (H_2_S) has long been viewed simply as a toxic gas with an odorous smell. Its dangerous properties were recognized as far back as the 18th Century when cesspit workers exposed to high environmental levels of H_2_S developed eye inflammation and bacterial infection [1] (Figure 1). More recently, however, H_2_S was discovered to exist endogenously and has emerged as an omnipotent signaling molecule, specifically in the cardiovascular system [2–7]. Several years ago, cardiovascular researchers largely focused on the other gaseous signaling molecules, nitric oxide (NO) and carbon dioxide (CO). Consensus formed that NO and CO based therapies protect the brain, heart, and circulation against a number of cardiovascular diseases [8–14]. Because endogenously produced H_2_S is a gaseous signaling molecule capable of regulating physiological processes (similar to NO and CO), we investigated its potential role as a cardioprotective agent. Our group has shown specifically that H_2_S protects against myocardial ischemia/reperfusion (MI/R) injury and preserves cardiac function following the onset of heart failure in various preclinical model systems.

## 2. Endogenous Synthesis of Hydrogen Sulfide in Mammals

Experimental studies reveal that H_2_S is produced at nano- to micromolar levels both enzymatically and nonenzymatically [15]. The continuous enzymatic production is critical due to the extremely short biological half-life of the molecule (estimated to be between seconds to minutes) [16, 17]. Nonenzymatic H_2_S can form via the reduction of thiol-containing molecules when H_2_S is released from sulfur stores such as sulfane sulfur. Two H_2_S producing enzymes are part of the cysteine biosynthesis pathway: cystathionine gamma lyase (CSE) and cystathionine beta synthase (CBS). These enzymes coordinate with L-cysteine to produce H_2_S, L-serine, pyruvate, and ammonia [2, 4]. Originally, the endogenous production of H_2_S in the brain was attributed to CBS [18]. However, more recently, the third enzyme, 3-mercaptopyruvate sulfurtransferase (3-MST), was reported to manufacture roughly 90% of H_2_S in the brain and is largely concentrated in the mitochondria [19]. 3-MST produces H_2_S from *α*-ketoglutarate and L-cysteine via metabolic actions with cysteine aminotransferase and glutamate [19]. The distribution and function of CBS, CSE, and 3-MST under normal physiological conditions remain controversial and unclear. However, we have found that all 3 enzymes are expressed in the heart [20] and a global genetic deletion of CSE (global CBS and 3MST KO mice have not yet been reported) results in significant reductions in myocardial and circulating H_2_S and sulfane sulfur levels [21]. As this field advances, more discoveries will likely unfold and give us more insight into the physiological mechanism of these enzymes.

## 3. Hydrogen Sulfide and Myocardial Infarction

Myocardial infarction remains a leading cause of mortality worldwide [22]. It is well established that myocardial ischemia/reperfusion (MI/R) injury stimulates tissue destruction and often leads to heart failure [23]. While reperfusion relieves ischemia, it also results in a complex reaction that leads to cell injury caused by inflammation and oxidative damage [24]. In the first study, to establish an* in vivo* model for MI/R in mice, the left coronary artery (LCA) was transiently ligated and reperfusion followed by removal of the ligating suture [25]. Following 30 minutes of ischemia, mice were administered sodium sulfide (Na_2_S) (50 *μ*g/kg) into the left ventricle (LV) lumen. Mice receiving the donor at the time of reperfusion displayed a 72% reduction in infarct size compared to the vehicle treated mice [25]. Cardiac troponin-I (cTnI) evaluation, an additional marker for myocardial injury, also affirmed myocardial preservation in the H_2_S treated group. Additionally, LV echocardiographic analysis following 72 hours of reperfusion revealed that H_2_S treated mice displayed no increase in post-MI/-R LV dimensions (left ventricular end-diastolic dimensions and left ventricular end-systolic dimensions), while the vehicle treated group showed significantly increased wall thickening [25].

A subsequent study examined the impact of genetically modifying an enzyme responsible for much of endogenous H_2_S production (CSE) [25]. Using a heavy chain *α*MHC promoter in coordination with the cystathionine (Cth) gene sequence (responsible for CSE production), a cardiac specific transgene mouse was created to constitutively overexpress the CSE enzyme. These mice had a significantly elevated production rate of H_2_S, as expected, and were subjected to a similar MI/R protocol. Following 45 minutes of ischemia and 72 hours of reperfusion, the transgenic mice expressed significantly reduced infarct size compared to the wild-type group. These findings reveal that both exogenous donors and endogenously elevated H_2_S serve to protect against ischemia-reperfusion injury in the murine heart.

The mechanisms by which H_2_S protected against MI/R injury, we found, are through preservation of mitochondrial function, reduction of cardiomyocyte apoptosis, anti-inflammatory responses, and antioxidant effects that limit cell damage and death. Mitochondria are essential for cell survival and energy production. They are unique in that they regulate cell death and apoptosis and maintain oxidative phosphorylation following MI in a manner that helps to preserve myocyte survival [26].* In vitro *experiments revealed a dose-dependent reduction in oxygen consumption followed by a recovery to baseline levels in the H_2_S treated group [25]. Additionally, H_2_S at the time of reperfusion preserved function as noted by increases in efficiency of complexes I and II of the electron transport chain. In an ischemia setting, mitochondrial function can be compromised as a result of an increase in reactive oxygen species (ROS), which can lead to uncoupling and increased infarction [27, 28]. High doses of H_2_S can slow down cellular respiration by inhibiting cytochrome c oxidase, lowering metabolism into a protected, preconditioned state [29]. The inhibition of respiration has been shown to protect against MI/R injury by limiting the generation of ROS species [30, 31].

We also found H_2_S to have antioxidant properties mediated by Nrf-2 signaling. Nrf-2 is a potent antioxidant transcription factor that can translocate from the cytosol to the nucleus to induce various antioxidant proteins. This protein promotes oxidant defenses and reduces oxidative stress. When mice were treated with a long acting H_2_S donor, diallyl trisulfide (DATS), following acute MI, Nrf-2 translocated from the cytosol to the nucleus while overall levels of Nrf2 remained constant within the cell [32]. Additional studies further demonstrate the downstream signaling of Nrf2 induced by H_2_S to promote antioxidant defenses [33–36]. These cardioprotective actions, we believed, would also prove to be protective in other heart diseases. We then investigated H_2_S in heart failure.

## 4. Hydrogen Sulfide and Heart Failure

Heart failure is the heart's inability to sufficiently supply blood to meet the needs of the body. In the United States, it has become the most common discharge diagnosis in patients 65 years or older and treatments remain insufficient [37, 38]. Therefore, the investigation of therapeutic options to attenuate cardiac dysfunction in heart failure remains clinically relevant and critical.

**Figure 2:**
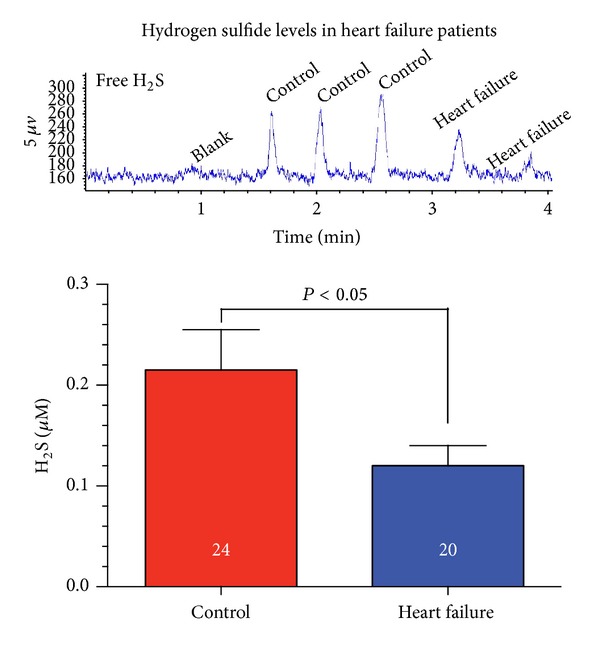
Circulating hydrogen sulfide levels are diminished in heart failure patients. We evaluated H_2_S levels in heart failure patients (*n* = 24) compared to age-matched control subjects (*n* = 20). Serum free H_2_S (*μ*M) levels were significantly reduced (*P* < 0.05) in heart failure patients. Serum samples were obtained from patients enrolled in the Atlanta Cardiomyopathy Consortium (TACC). This prospective cohort study enrolls patients from the Emory University-affiliated teaching hospitals, the Emory University Hospital and Emory University Hospital Midtown, and Grady Memorial Hospital in Atlanta. All patients undergo detailed medical history surveys, electrocardiogram, standardized questionnaires, and blood and urine sample collection at baseline. All patients provide written informed consent prior to enrollment. The Emory University Institutional Review Board has approved this study. H_2_S levels were measured in the blood according to previously described methods [20].

Our group found that heart failure patients have marked reductions in circulating H_2_S levels compared to age matched controls (Figure 2). In a recent study, Peter et al. reported elevated plasma H_2_S levels in patients with vascular disease [39]. The results in this study do not contradict our findings of reduced H_2_S in heart failure patients. The patient profiles in the two studies are dissimilar and do not represent similar disease states. The heart failure patients analyzed in the current study suffer from severe end stage cardiomyopathy with reduced heart function [40]. Conversely, patients in the recent study suffered from coronary or peripheral arterial disease. We do not take these findings as conflicting but acknowledge that changes in H_2_S are dependent on numerous factors, such as the type of cardiovascular disease (i.e., coronary heart disease or heart failure). The discovery of H_2_S deficiency in heart failure patients led to our exploration of H_2_S therapy for the treatment of heart failure. In our preliminary study to create a heart failure in the murine heart, transverse aortic constriction (TAC) between the brachiocephalic trunk and the left carotid artery produced a hypertrophic, pressure overload induced model [20]. We observed greater than a 60% decrease in both myocardial and circulating H_2_S levels following TAC compared to naïve mice. This finding was in accordance with our discovery that heart failure patients have a H_2_S deficiency. We next compared mice devoid of the CSE enzyme to wild type mice following TAC. CSE KO mice exhibited significantly greater cardiac dilatation and exacerbated dysfunction than wild-type mice, indicating the demand of H_2_S to protect against pressure overload heart failure. We then examined H_2_S therapy in the setting of heart failure. SG-1002, an H_2_S donor, was infused in the chow and was continuously administered throughout the study beginning the day of aortic constriction. Interestingly, the therapy prevented cardiac dilatation and preserved LV function throughout the 12-week course of the study. Morphological analysis after TAC revealed that H_2_S treated mice had minor cardiac enlargement compared to the vehicle group, indicating reduced hypertrophy. Similar analysis displayed less pulmonary edema in the H_2_S treated group.

In addition to its antioxidant actions and mitochondrial protection, H_2_S appears to promote angiogenic responses and inhibit fibrosis during heart failure. Histological analysis revealed that left ventricular intermuscular and perivascular fibrosis were significantly attenuated at 6 weeks following TAC in the H_2_S treated group [41]. Mice treated with H_2_S donors in the setting of heart failure also displayed significantly greater VEGF (a potent angiogenic cytokine) and CD31^+^ (an endothelial cell marker) expression in the myocardium.

Other studies have concurred that the downregulation of H_2_S is involved in the pathogenesis of cardiomyopathy induced by Adriamycin [42] and myocardial injury induced by isoproterenol [43]. In these studies, myocardial injury resulted in decreased CSE activity, reduced heart and plasma H_2_S levels, and increased oxidative stress. However, total CSE gene expression was elevated in the heart failure models. These findings were in accordance with our pressure overload induced heart failure model where we observed a robust CSE protein expression but a significant decrease in blood and myocardial H_2_S levels compared to sham mice [20].

## 5. Mechanisms of Cardioprotection

**Figure 3:**
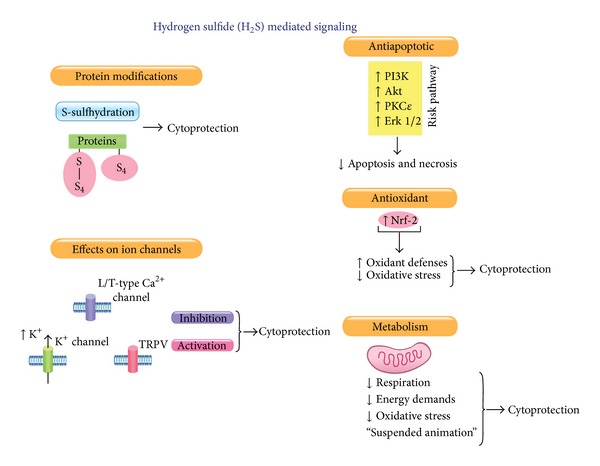
Hydrogen sulfide cardioprotective signaling. H_2_S is known to modify proteins (s-sulfhydration), to modify the function of various ion channels (i.e., Ca^2+^, K^+^, and TRPV), to mitigate apoptosis and oxidative stress, and to be a potent modulator of cellular metabolism.

Many of the cardioprotective mechanisms resulting from H_2_S therapy in acute MI and congestive heart failure are similar (Figure 3). For example, H_2_S promotes the translocation of the nuclear transcription factor, Nrf2, from the cytosol to the nucleus resulting in the subsequent expression of numerous detoxifying genes such as heme oxygenase 1 (HO-1), superoxide dismutase, and catalase [44, 45]. In addition, H_2_S protects cells against oxidative stress by increasing glutathione levels in a cysteine dependent manner [46]. Although H_2_S acts independently to activate antioxidant and prosurvival signals, crosstalk between H_2_S and NO may also play an important role [21, 47]. H_2_S is known to activate endothelial nitric oxide synthase (eNOS) and augment NO bioavailability [20, 41]. NO is well established as a signaling molecule with antioxidant characteristics [48, 49] and may enhance these protective signaling actions.

H_2_S also plays a critical role in the protection of mitochondria during ischemic states in a manner that significantly attenuates cell death and apoptosis [26, 50]. Following MI/R injury, H_2_S treated mice exhibited diminished activation of caspase-3 and a decreased TUNEL positive nuclei count [25]. H_2_S also promotes antiapoptotic signaling pathways by altering p38, Erk 1/2, and PI3K expression [51, 52]. Acutely, H_2_S attenuates mitochondrial respiration to induce a “suspended-animation-” like state and reduces cellular respiration and oxygen demand [29, 53]. Establishing this state can preserve mitochondrial function by reducing oxidative stress and mitigating apoptotic signaling. This renders H_2_S particularly protective against myocyte injury in settings such as acute MI/R.

One of the earliest proposed benefits of H_2_S as a physiological modulator on the vasculature is its ability to prevent inflammation [6, 7, 54]. H_2_S prevents leukocyte adhesion to the vessel wall and inhibits the expression of adhesion molecules [55]. Moreover, in naïve animals, H_2_S has promoted vessel growth and suppressed antiangiogenic factors [56, 57]. H_2_S has also been shown to decelerate the progression of cardiac remodeling and promote angiogenesis in a congestive heart failure [20, 41]. Angiogenesis is a complex biological process that involves extracellular matrix remodeling and endothelial growth, migration, and assembly into capillary structures [58]. Decompensated heart failure is associated with a decline in vascular growth and reduced blood flow [57], so H_2_S may be an attractive therapeutic option for the treatment of the progression of heart failure.

## 6. Future Directions

 A number of laboratories have clearly demonstrated the cardioprotective actions of H_2_S in both acute myocardial infarction and heart failure [59–62]. The mechanisms responsible for these protective effects include the downregulation of oxidative stress responses, modulation of mitochondrial respiration, attenuation of apoptosis, and increasing vascular growth and angiogenesis. H_2_S is known to activate multiple and diverse pathways simultaneously and exhibits cross-talk with the NO and CO signal pathways to amplify a cytoprotection response. In addition, H_2_S freely circulates throughout the body, diffuses across cellular membranes, and acts on multiple cellular targets [63]. Furthermore, the actions of H_2_S are not limited to the heart muscle alone but can impact the entire cardiovascular system including blood vessels [7]. In fact, with this field only recently developing, there are tremendous opportunities for further discovery relating to H_2_S physiology, pharmacology, and pathology. Recent experimental data provide evidence that H_2_S can prevent atherosclerosis and promotes angiogenesis in the peripheral arteries [55, 64]. This may prove beneficial when treating vascular diseases that demand collateral vessel growth such as peripheral artery disease (PAD) and critical limb ischemia (CLI). Recently, several groups have reported that H_2_S also plays a role in pulmonary hypertension and acute lung injury [65, 66]. Although H_2_S does not have the potent vasodilation capabilities of NO, the combination of vascular smooth muscle relaxation and potent antioxidant properties may be the source for protection against pulmonary hypoxia and hypertension. In both the liver and the kidneys, H_2_S is a protective preconditioning agent against ischemia/reperfusion injury [67, 68]. Similarly to myocardial ischemia/reperfusion protection, H_2_S protects by its ability to mitigate apoptosis and modulate oxidative stress.

Discovering the most effective H_2_S donors is also a challenge facing the field. Drugs such as NaHS, Na_2_S, and GYY4137 are all effective H_2_S donors, but their rapid half-life renders them less effective for treating chronic diseases. The slow releasing polysulfides deliver a more gradual release of H_2_S [32]. Other proposed sulfide-modulating agents such as S-propargyl-cysteine do not substantially raise H_2_S levels* in vivo* [69]. Dietary formulations, such as SG-1002, can be used as medical foods to replenish an H_2_S deficiency that may occur from diseases such as heart failure. Because of the short half-life of H_2_S (estimated to be between seconds and minutes [17, 70]), developing a drug with specific on-site (organ or organelle specific) delivery would also be beneficial.

Following in the footsteps of nitric oxide and carbon monoxide, hydrogen sulfide is rapidly emerging as a critical cardiovascular signaling molecule. Although the complete actions of this gas remain under investigation, the therapeutic options relating to cardiovascular disease are extremely promising. The coming years or research will dictate the means of utilizing this molecule effectively against various cardiovascular disease states.
